# Learning from a crisis: a qualitative study of the impact on mothers’ emotional wellbeing of changes to maternity care during the COVID-19 pandemic in England, using the National Maternity Survey 2020

**DOI:** 10.1186/s12884-022-05208-7

**Published:** 2022-11-23

**Authors:** Jenny McLeish, Sian Harrison, Maria Quigley, Fiona Alderdice

**Affiliations:** NIHR Policy Research Unit in Maternal and Neonatal Health and Care, National Perinatal Epidemiology Unit, Nuffield Department of Population Health, University of Oxford, Oxford, UK

**Keywords:** COVID-19, Maternity care, Mothers' experiences, Emotional wellbeing

## Abstract

**Background:**

Pregnancy and the postnatal period can be times of psychosocial stress and insecurity, but high quality maternity care and social support can help mothers cope with stress and feel more secure. The COVID-19 pandemic and associated social and economic disruption increased rates of antenatal and postnatal stress, anxiety and depression, and also had profound impacts on the organisation of maternity services in England.

**Methods:**

This was a qualitative descriptive study of the impact of pandemic-related changes to maternity care on mothers’ emotional wellbeing, using inductive thematic analysis of open text responses to the National Maternity Survey (NMS) 2020 in England. A random sample of 16,050 mothers who gave birth 11-24^th^ May 2020 were invited to take part in the survey, and 4,611 responded, with 4,384 answering at least one open text question.

**Results:**

There were three themes: ‘Chaos: impact of uncertainty’, ‘Abandoned: impact of reduction in care’, and ‘Alone: impact of loss of social support’. Mothers valued maternity care and many experienced additional stress from chaotic changes and reduction in care during the pandemic; from health professionals’ own uncertainty and anxiety; and from restrictions on essential social support during pregnancy, labour and birth. Others felt that health professionals had communicated and cared for them well despite the changes and restrictions, and these mothers felt psychologically safe.

**Conclusions:**

Planning for future crises should include considering how necessary adaptations to care can be implemented and communicated to minimise distress; ensuring that mothers are not deprived of social support at the time when they are at their most vulnerable; and supporting the psychological welfare of staff at a time of enormous pressure. There are also lessons for maternity care in ‘normal’ times: that care is highly valued, but trust is easily lost; that some mothers come into the maternity system with vulnerabilities that can be ameliorated or intensified by the attitudes of staff; that every effort should be made to welcome a mother’s partner or chosen companion into maternity care; and that high quality postnatal care can make a real difference to mothers’ wellbeing.

## Background

Having a baby is a major life transition characterised by insecurity and psychological vulnerability [1–3]. Maternal stress can be caused during pregnancy by changes in the mother’s body, identity, and relationships, along with fears about childbirth, the child’s health, and approaching parenthood [4, 5]. Postnatal stress may arise from feeling unable to cope with the practical and psychological challenges of early motherhood [6, 7]. Lack of access to maternity care is associated with increased stress [8], but poor quality maternity care that does not meet the needs of the individual mothers can in itself become an additional source of stress [7]. Perinatal stress has been associated with a range of adverse maternal and neonatal outcomes, including increased depression and anxiety [5, 9, 10].

Social support, which is defined as a person’s perception of the availability of others to provide emotional, psychological and material resources, can ‘buffer’ the impact of stress on an individual [11]. When a mother is experiencing stress and insecurity, social support can enhance her ability to cope [12, 13]. Pregnant women and new mothers may receive social support from their partner, family and friends, and created networks of mothers, as well as medical and social support from health professionals [7, 14–18]. Women’s sense of security at birth and afterwards is strongly affected by the presence and supportive behaviours of her partner and a caring midwife [3].

The COVID-19 pandemic increased psychosocial stress, uncertainty, and rates of self-reported depression and anxiety for pregnant women and new mothers, due to social, economic, and healthcare disruptions, as well as a lack of evidence regarding the effect of COVID-19 infection on the unborn child [19–24]. In England, national ‘lockdown’ rules prohibited face-to-face social contact with people from outside the immediate household from late March to June 2020, in November 2020, and from January to March 2021. Local lockdowns were in force in different parts of the country with high COVID-19 rates at other times. Pregnant women were classified as ‘clinically vulnerable’ to COVID-19 and were advised to be particularly stringent in observing social distancing, but were confused about what this meant in practice [25]. Family finances were affected by job loss and furlough (temporary suspension), and rates of domestic abuse increased [20, 26].

At the same time there were dramatic but inconsistent alterations in the maternity and postnatal services offered in different National Health Service (NHS) areas, including a reduction in the options for place of birth (withdrawing home births and midwifery-led units); reduction in the number of antenatal and postnatal appointments; replacement of face to face appointments with telephone or video calls; suspension of antenatal classes; withdrawal of continuity of midwifery carer; closure of health visitors’ drop-in clinics where babies could be weighed and checked; and cancellation or reduced access to other services such as breastfeeding support [24, 27]. Restrictions directly affecting social support included prohibitions on the presence of a mother’s partner or other companion, who were (1) not allowed to attend her antenatal appointments and scans or to be with her if she was admitted to hospital antenatally; (2) not allowed to be with her during labour in a hospital or birth centre until she was confirmed to be in established labour, and not allowed to be present at birth if the mother had symptoms of COVID-19; and (3) required to leave as soon as she was transferred to the postnatal ward and not allowed to visit [28]. Similar changes to maternity care in Canada were found to be significantly associated with an increase in depression and anxiety [29], and changes to antenatal appointments were associated with increased maternal stress in the USA [22], consistent with findings about maternal distress during previous outbreaks of infectious disease [30].

During natural disasters, it has been found that social support and continuity of midwifery carer buffered women against the effects of subjective stress on postnatal depression and anxiety [31, 32]. This highlights the importance of identifying ways of protecting and enhancing the stress-buffering potential of maternity care during any crisis, and reducing ways in which it may exacerbate mother’s stress either directly or indirectly by obstructing social support; this learning could also be used to inform high quality care during ‘normal’ times. The National Maternity Survey 2020 [24], commissioned and planned before the pandemic, was sent to a random population-based sample of women who gave birth in England during a two week period in May 2020, so it captured women’s experiences of maternity care during the first period of COVID-19 restrictions. The survey’s quantitative findings showed that the great majority of respondents were satisfied with their antenatal care (84%) and intrapartum care (85%), and half were satisfied with their postnatal care (53%). In this study, we used the open text responses to the National Maternity Survey to investigate how new mothers described the ways in which the pandemic-related changes to maternity and postnatal care affected their emotional wellbeing. By using the extreme example of maternity care during a crisis, this study seeks both to illuminate the ways in which maternity care can enable and enhance mothers’ ability to cope with the stress of having a baby in ‘normal’ times, and to identify lessons for protecting maternal wellbeing in future crises.

## Methods

### Study design

This was a qualitative descriptive study based on open text responses to the National Maternity Survey 2020. A qualitative descriptive design aims to explore participants’ experiences and perceptions, staying close to the data while acknowledging the role of both participants and researchers in the production of knowledge [33]. Data analysis and interpretation were theoretically informed by phenomenological social psychology, which focuses on participants’ lived experiences and the subjective meanings of social interactions [34].

### Data collection and dataset used

The National Maternity Survey 2020 was a cross-sectional survey, with a random sample of 16,050 women identified by the Office for National Statistics using birth registration records. The women were aged 16 years or older, living in England at the time the birth was registered, and had given birth to their baby in England between 11 and 24^th^ May 2020; they were invited by letter to take part in the survey six months after they had given birth. The full survey methodology has been reported by Harrison et al. [24]. 4,611 women responded to the National Maternity Survey (a response rate of 28.9%). 4,384 women answered at least one of the nine open text questions on which this study is based, covering antenatal, intrapartum and postnatal care (see Table 1). The length of individual respondents’ combined open text answers ranged from a few words to over 2,500 words, with 738 respondents writing over 250 words.

**Table 1:** Open text questions used in the analysis

1. Are you aware of any changes to the care you received during your pregnancy because of COVID-19? If yes, please tell us more
2. Is there anything else you would like to tell us about your pregnancy or the care you received?
3. Please use this space to tell us about any changes to your plans for birth and how well informed you felt about any changes
4. Some hospitals placed restrictions on partners/birth partners attending births. Did you/your partner/birth partner face any restrictions? If yes, please briefly describe the impact of any restrictions
5. Is there anything else you would like to tell us about your labour or the birth of your baby?
6. Is there anything else you would like to tell us about your own or your baby’s postnatal care?
7. Do you feel the amount of support you can access has changed because of COVID-19? If yes, please tell us more
8. Is there anything else you would like to tell us about yourself, your baby or the care you have received?
9. Were there other important questions that you feel we should have asked? If yes, please tell us more

### Data analysis

Survey responses were analysed using inductive thematic analysis [35]. To gain initial familiarity with the overall data, the answers to antenatal questions (Q1-2), intrapartum questions (Q3-5), and postnatal and general questions (Q6-9) were first read separately. Each individual respondent’s answers to all of these nine questions were then merged into a single cell on an Excel spreadsheet, along with some socio-demographic details: current age, previous births, country of birth and ethnicity. This spreadsheet was uploaded to NVIVO to record inductive coding, which was not focused on maternity and postnatal experiences per se, but on women’s spontaneous descriptions of how these experiences had made them feel. Codes were refined during the analysis, and were developed into themes.

To make thematic analysis of this large dataset more feasible, detailed coding was undertaken of half of the responses (*n* = 2,192), and an initial thematic structure was developed based on these codes. This thematic structure was then checked against the remaining half of the responses (*n* = 2,192), with a particular attention to looking for responses that might disconfirm or refine the initial thematic findings. To ensure that the analysis foregrounded the experiences of women who might be more likely to experience disadvantage because of ethnicity, migration or age, selection of the responses for detailed coding followed this sequence:

- 1. All women of any age who identified their ethnicity as Black, Asian, mixed/multiple and other ethnic minorities (*n* = 589).
- 2. All women of any age who identified their ethnicity as Other White (*n* = 406).
- 3. All women who identified their ethnicity as White British or did not state ethnicity, and were aged under 25 (*n* = 264).
- 4. Women who identified their ethnicity as White British or did not state ethnicity, and were aged 25 and older, ordered by the date in which they responded to the survey (*n* = 933).

Longer responses were not prioritised over shorter ones because this might have given less prominence to the experiences of mothers with lower literacy or English language skills. To increase the validity of the analysis, one researcher analysed all of the data and the second researcher independently analysed a subset; codes and developing themes were discussed and agreed. Both researchers approached the analysis reflexively, acknowledging the potential impact of their existing knowledge as experienced researchers in this field and of personal perspectives as White, UK-born women with children born before the pandemic. A final step was selection of quotations to illustrate the themes and subthemes, guided by demographic variables, to give voice to women from a range of backgrounds. Spelling was standardised and grammar was edited if this was necessary to make the meaning clear.

## Results

### Participants

Socio-demographic characteristics of the mothers who gave open text answers are shown in Table 2.

**Table 2:** Socio-demographic characteristics of mothers who gave open text answers

	**Number of mothers (*n* = 4384)**	**Percentage**
**Ethnicity**		
Asian	314	7.2
Black	107	2.4
Mixed/multiple ethnicity	93	2.1
White British	3365	76.8
Other White	409	9.3
Other	47	1.1
Not stated	28	0.6
**Country of birth**		
UK	3554	81.1
Outside UK	800	18.2
Not stated	30	0.7
**Age in years**		
16–19	23	0.5
20–24	286	6.5
25–29	916	20.9
30–34	1712	39.1
35–39	1129	25.8
40 +	290	6.6
Not stated	28	0.6
**IMD quintile**		
1 (most deprived)	635	14.5
2	821	18.7
3	924	21.1
4	1024	23.4
5 (least deprived)	980	22.4
**Age on leaving education in years**		
< 16 years	473	10.8
17–18 years	1165	26.6
19 + years	2708	61.8
Not stated	38	0.9
**Married/partnered**		
Yes	3973	90.6
No	411	9.4
**Previous birth**		
No	2249	51.3
Yes	2045	46.6
Not stated	90	2.1

### Thematic findings

**Fig. 1:**
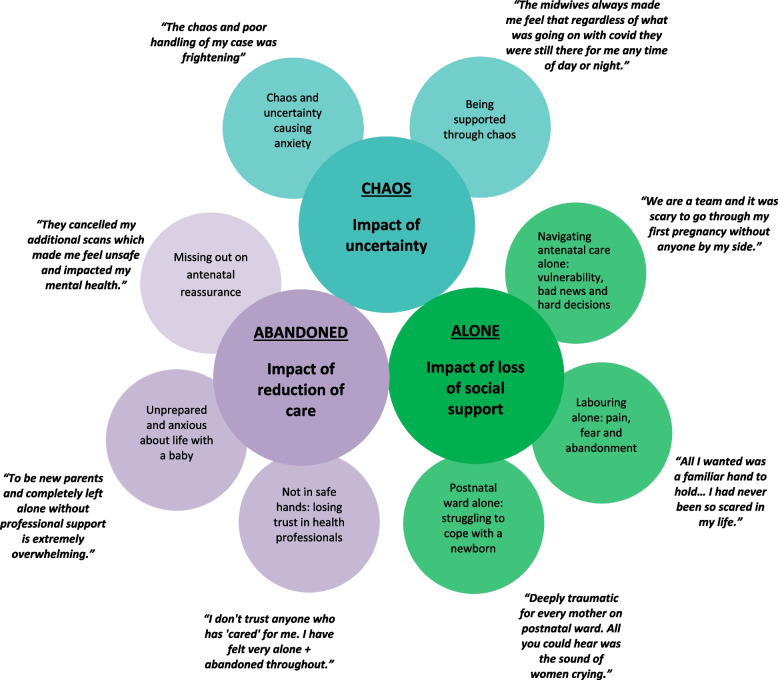
Themes, subthemes and example quotations

Three themes were identified related to the emotional impact of the changes introduced to maternity care: ‘Chaos: impact of uncertainty’, ‘Abandoned: impact of reduction in care’, and ‘Alone: impact of loss of social support’. These themes and their subthemes are shown in Fig. 1. Although the data analysis was purposively segmented by ethnicity and age, there were no differences by ethnicity or age in the themes identified. The only thematic differences identified were between mothers who were having their first baby and mothers who already had a child. Many first time mothers commented on their additional vulnerability to stress, and many experienced mothers said that they would not have coped if this had been their first child.

### Theme 1 CHAOS: impact of uncertainty

This theme describes how mothers experienced the chaos and uncertainty surrounding the changes to their maternity care during the first wave of COVID-19, and how the professional response could add to their anxiety or help to mitigate it.

#### Subtheme 1.1 Chaos and uncertainty causing anxiety

Mothers described a maternity system that had become chaotic and unpredictable as varying restrictions were introduced or lifted, and scheduled appointments were cancelled without notice, with inconsistent rules between different providers. Many said that the latter part of their pregnancies had been dominated by worrying about whether their partner would be present at birth, and whether they would be able to give birth at the place and in the manner they had chosen.

“The emotional stress and worry of not knowing how your pregnancy or birth plans would be affected caused many tears and sleepless nights for myself and my partner. The hospital couldn't provide answers as they said the rules were ever changing therefore we wouldn't know if my partner could be there until maybe the day before. The whole experience, lack of communication and knowledge around covid and care was overwhelming.” (Age: 34; Previous birth: Yes; Born: UK; Ethnicity: White & Black Caribbean).

For some mothers, anxiety had been increased by what they perceived as haphazard and inadequate communication of the changes to care, with no certainty that they knew the up-to-date position or where to find this information.

“I found out that my antenatal midwife appointments were cancelled through Facebook. I spent the last 10 weeks of pregnancy sick with worry—that my husband would not be allowed at the birth. The communication on this was terrible. This level of stress should not be put on pregnant women.” (Age: 34; Previous birth: Yes; Born: UK; Ethnicity: White British).

Many mothers used phrases like *‘we were forgotten’* to describe the feeling that there was no longer any coherent plan for their antenatal and postnatal care. This caused them anxiety as they were not confident that there was a system capable of keeping them safe.

“There was a lot of unknown, and changes to plans last minute which was very anxiety-invoking …. My midwife was put back on the wards and I had no appointments at all. No one seemed to know who my midwife was or when my next appointment would be.” (Age: 35; Previous birth: Yes; Born: Ireland; Ethnicity: Other White).

Although there were some general comments about how the absence of continuity of midwifery carer had inhibited the development of trust between mothers and their midwives, this was particularly problematic where specialist care or continuity of carer had been started but then withdrawn. This had intensified mothers’ fear that no one was looking out for them or able to support them through the uncertainty.

“Antenatal care with specialist midwife team pre-Covid was fantastic. When Covid reached the UK, the chaos and poor handling of my care was frightening … No further contact with my named midwife and her team, no option for home birth, no access to the 24/7 number to call.” (Age: 34; Previous birth: No; Born: UK; Ethnicity: White & Asian).

Sometimes it was health professionals’ own difficulties in coping with the pressure of pandemic changes that lead to mothers losing confidence in their maternity carers.

I expressed anxiety over Covid to one midwife who proceeded to tell me how hectic it was and that she was on medication for stress to help deal with the pressure. The phone call made me feel worse.” (Age: 30; Previous birth: No; Born: UK; Ethnicity: White British).

#### Subtheme 1.2 Being supported through chaos

By contrast, there were also mothers who felt that despite the chaotic situation, they could trust the maternity team to minimise disruption and to continue to protect their safety and that of their babies through high quality care.

“Even though there was Covid I never missed any appointment, I have got all the care I needed, so thank you so much to the doctors, nurses, midwives & health visitors. I am grateful & appreciated all their hard work. Thank you so much for NHS.” (Age: 38; Previous birth: No; Born: Ethiopia; Ethnicity: Black African).

Some mothers focused their appreciation on the efforts staff had made to minimise uncertainty by communicating changes clearly.

“I was very satisfied with the service provided even in the hardest time of COVID-19. My midwife informed me fully what to expect & deal with it so I was assured.” (Age: 32; Previous birth: Yes; Born: Pakistan; Ethnicity: Pakistani).

Mothers who were already anxious about the uncertain risks from COVID-19 particularly appreciated health professionals who took time to address their concerns.

“Midwife are angels, they really are. They did everything possible to make me feel reassured in this hard time.” (Age: 26; Previous birth: Yes; Born: UK; Ethnicity: White British).

Sometimes this appreciation was linked to a perception that health professionals had gone above and beyond their professional duty, which made mothers feel cared for and safe.

“The midwife took extra care to look after me during the pandemic. Even though they couldn’t be close enough, they still listen to what I wanted. I was very well treated.” (Age: 26; Previous birth: Yes; Born: Guinea; Ethnicity: Black African).

For other mothers their feeling of safety came from an experience of receiving truly personalised care that overrode the changes to care or restrictions.

“Although we were in full lockdown the community midwife I spoke to on the phone on my first full day at home recognised that I was not coping and came to see me at home. Most of the midwife appointments that were meant to be over the phone were done face to face which was very reassuring, and the midwives always made me feel that regardless of what was going on with covid they were still there for me any time of day or night.” (Age: 35; Previous birth: No; Born: UK; Ethnicity: not stated).

### Theme 2 ABANDONED: impact of reduction of care

This theme describes how mothers’ experienced the reduction of professional support antenatally and postnatally, and the difficulty of contacting health professionals when their advice was needed, as increasing their stress and anxiety. Many mothers said they felt *‘abandoned’, ‘ignored’, ‘cast aside’, ‘neglected’* or *‘left to our own devices’* by the health professionals who they had expected to rely on for information, advice and clinical care.

#### Subtheme 2.1 Missing out on antenatal reassurance

Many mothers commented that the reduction of professional support antenatally had made them worry that pregnancy problems might be overlooked. When face-to-face care was replaced with telephone appointments, they specifically missed the reassurance of regular testing of blood pressure and urine to identify maternal health conditions, and checks on the baby’s heartbeat, position and growth.

“Felt like I was very much left on my own towards my pregnancy. I didn't have regular blood pressure checks or urine checks which led to massive anxiety around the health of me and my baby.” (Age: 25; Previous birth: No; Born: UK; Ethnicity: White British).

Mothers said they found the loss of face-to-face appointments, including these checks and planned extra scans, particularly stressful where there were pregnancy complications or concerns.

“They cancelled my additional scans which made me feel unsafe and impacted my mental health. I had a high risk pregnancy.” (Age: 36; Previous birth: Yes; Born: Ireland; Ethnicity: Other White).

As well as the change in format, mothers commented that appointments felt rushed and superficial, creating additional stress when health professionals did not answer their questions.

“Some of my appointments were very rushed and did not give me ample opportunity to ask questions / seek advice…I found some of these rushed sessions quite stressful and anxiety inducing.” (Age: 33; Previous birth: No; Born: Ireland; Ethnicity: Other White).

#### Subtheme 2.2 Unprepared and anxious about life with a baby

Some first time mothers described feeling unprepared for birth and motherhood, because they had been expecting to get information they could trust from antenatal classes, which had been cancelled. Some of their comments were about being prepared for labour and understanding birth options, but most were about how the lack of advance information had contributed to a lack of parenting confidence and consequent stress.

“I was completely clueless on so many things that I was heavily relying to learn about in my classes … There were so, so, so many questions and I felt so confused.” (Age: 26; Previous birth: No; Born: United States; Ethnicity: Other White).

After birth, many mothers were shocked at the lack of postnatal support in the community, and linked this to their lack of parenting confidence.

“To be new parents and completely left alone without professional support is extremely overwhelming (not to mention during a global pandemic) … It has definitely triggered levels of anxiety and isolation in me that I haven't felt before.” (Age: 32; Previous birth: No; Born: UK; Ethnicity: White British).

Without access to advice from health professionals and to weighing clinics, many mothers became anxious about whether they were making good decisions about caring for their baby including feeding and recognising signs of illness. Some reported that their babies had indeed developed undiagnosed health conditions including jaundice, and feeding problems resulting in hospital admission. They specifically missed the reassurance of knowing whether their babies were gaining weight appropriately, and also worried if their own physical recovery was not straightforward, with some experiencing serious health consequences such as undiagnosed infections in their caesarean wound, perineal tear or episiotomy site. Many mothers commented on the stress of not knowing what was *‘normal’* for a baby and their own physical and mental health, and being ‘*left to figure out things on our own’*.

“I would love to have my baby checked more often. I don't know if I am doing well or not…I often cry and feel I have lost myself.” (Age: 34; Previous birth: No; Born: Italy; Ethnicity: Other White).

“There hasn't been any [postnatal care]. All I have been told is go to A&E if anything is unusual. This is my first baby so I don't know what is unusual or not.” (Age: 37; Previous birth: No; Born: UK; Ethnicity: White British).

Mothers’ descriptions of the intense isolation and strain they felt during periods of ‘lockdown’ are outside the scope of this paper. However, postnatal advice from health professionals was particularly missed by first-time mothers who were cut off from family social support by distance or by legal restrictions on contact with other households because of COVID-19.

“My family is all in [another country], I feel so alone, no one to help me out, I'm a young first time mum and anywhere professionally I have seeked help I have been either told to go elsewhere or been ignored entirely and due to COVID-19 I haven't been able to visit my family as often and receive help.” [Age: 22; Previous birth: No; Born: UK; Ethnicity: White British].

It should be noted that there were also a few mothers (mostly, but not only, mothers who already had a child) who said specifically that their confidence had not been negatively affected by the changes to care, and a few who said they felt the altered care was an improvement.

“Care received after birth was minimal. I felt I was just left to get on with it, but I felt happy and confident to do so.” (Age: 33; Previous birth: No; Born: UK; Ethnicity: Indian).

“Streamlining of appointments so I didn't have to go in as often—very sensible and probably could have been in place before COVID-19!” (Age: 37; Previous birth: Yes; Born: UK; Ethnicity: Other Mixed/Multiple).

#### Subtheme 2.3 Not in safe hands: losing trust in health professionals

Many mothers described appointments as having become much briefer, and commented that their care had felt like ‘a tick-box exercise’ as health professionals went through it formulaically and with minimal interaction. For some, this haste had communicated a lack of care which undermined feelings of safety in both antenatal and postnatal appointments.

“I felt like the midwife appointments were rushed and I was a number on a conveyer belt … She ran late one day and made myself and my partner feel as though we were an inconvenience.” (Age: 36; Previous birth: No; Born: UK; Ethnicity: Chinese).

Some mothers with mental health difficulties described trying to reach out for support both antenatally and postnatally, only to be ignored by health professionals who appeared to be unwilling to take the time to listen:

“I found lockdown very hard and felt severely depressed. I tried to speak to my midwife about this twice but she didn't seem to want to know, and wanted me out of the team as quick as possible.” (Age: 29; Previous birth: Yes; Born: UK; Ethnicity: White British).

Some mothers described how they had not felt able to build up a trusting relationship with health professionals in the absence of face-to-face appointments.

“There is nowhere to go to speak with someone in person due to covid. I am not one to share feelings over a phone and don’t feel you can grasp one’s feelings without seeing them.” (Age: 30; Previous birth: No; Born: UK; Ethnicity: White British).

Another reason for loss of trust was when mothers perceived their encounters with health professionals to have been superficial or inadequate.

“I haven't sought help or support for feeling down because I don't trust anyone who has 'cared' for me. I have felt very alone + abandoned throughout … the experience has made me wary about future pregnancies.” (Age: 29; Previous birth: No; Born: UK; Ethnicity: White British).

Some mothers said that their experience of the health visiting service as *‘useless’* or *‘non-existent’* had led them to conclude that their support was not worth having: if the system saw fit to withdraw this support from new mothers, it could not be as important as they had been led to believe. As a result, some mothers said that they would turn down future health visitor contacts or had already done so.

“There would ordinarily be a weekly clinic where you can speak with a health visitor and get baby weighed … My baby has not been weighed since he was 2 weeks. Makes you wonder are those appointments not important at all? If not then why so much emphasis pre-COVID?” (Age: 33; Previous birth: Yes; Born: Pakistan; Ethnicity: Pakistani).

"I had a health visitor schedule to come and see me at home 14 days after my baby was born. She did not turn up for the appointment and no explanation was given. I received a letter when my baby was 27 weeks old to arrange a visit from the same health visitor. I declined—any support I may have needed immediately after birth was not required at 27 weeks post-birth.” (Age: 36; Previous birth: Yes; Born: UK; Ethnicity: White British).

### Theme 3 ALONE: impact of loss of social support

This theme describes the impact on mothers of not being allowed to have their partner or other supporter (usually their mother) with them during their antenatal care, induction of labour, part or all of labour (if in a hospital or birth centre), and on postnatal wards. It explores the ways in which this loss of emotional and practical support interacted with the limitations on the support that health professionals offered mothers, in part because of the impact of COVID-19 on the staffing levels of maternity services.

#### Subtheme 3.1 Navigating antenatal care alone: vulnerability, bad news and hard decisions

When pregnant women were not allowed to have anyone accompany them to antenatal appointments, many said that they had missed the emotional support in dealing with an unfamiliar and stressful situation, especially if they lacked confidence in speaking to health professionals.

“Not having my partner in my appointments made me even more worried as we are a team and it was scary to go through my first pregnancy without anyone by my side.” (Age: 32 Previous birth: No; Born: UK; Ethnicity: Black Caribbean).

Some mothers said that they had found it difficult and distressing to make major decisions about maternity care without their partner’s support, and that this could leave a residual disappointment of having been pressurised into a decision by professionals.

“My husband was not able to be with me when issues were raised and doctors informed me I had to stay in hospital to be induced… Making decisions without my husband there was the hardest and most upsetting thing to do.” (Age: 30; Previous birth: No; Born: UK; Ethnicity: White British).

In the context of antenatal care, being separated from a partner or other companion was particularly distressing for mothers when there was ‘bad news’ at the appointment:

“Horrible, had to attend few appointments on my own and cry on the phone with the news received or having to call up for help to make a decision. Not having someone there to hold your hands and support.” (Age: 38; Previous birth: Yes; Born: Brazil; Ethnicity: Other Mixed/Multiple).

This stress was acute for mothers who attended an emergency appointment because of concerns about their baby’s wellbeing, and also mothers with complex pregnancies or histories of previous pregnancy loss, for whom ‘bad news’ felt like a real possibility each time.

“My pregnancy was a high risk pregnancy and I did not know when I was going to go into labour or even if my baby was going to survive. I had weekly appointments which my partner couldn’t attend due to Covid, and each week was nerve-wracking as we were having scans to check for a heart beat, so this was very distressing having to go alone.” (Age: 27; Previous birth: No; Born: UK; Ethnicity: White British).

#### Subtheme 3.2 Labouring alone: pain, fear and abandonment

Many women described their distress at having to labour alone without the support of their chosen birth partner, either until they were assessed as being established labour (usually defined by the hospitals as at least 4 cm dilation of the cervix) or in some cases throughout their labour and birth.

“There was nobody there to comfort me and tell me that everything will be alright or even walk me to the toilet. I was very distressed, anxious, scared. I just wanted to cry and kept begging midwife to allow my partner with me. It was very difficult to cope with pain and not having him there to comfort me.” (Age: 28; Previous birth: No; Born: Poland; Ethnicity: Other White).

Women’s descriptions of how they felt labouring alone repeatedly emphasised emotions such as *‘frightened’*, *‘scared’* and *‘terrified’*. As well as missing the emotional and practical support of a birth partner, they were frightened by being alone and in pain they couldn’t deal with, and by being unable to attract the attention of busy staff to help. Some women described the psychological impact of being without support during labour in very strong terms, for example as the *‘worst time of my life’*, and many used the word *‘traumatic’* to express the profound nature of this impact.

“I was completely alone for the first half of labour which was early—terrified. I couldn't reach my phone or midwife call button. The cleaner found me on the floor in my room.” (Age: 32; Previous birth: No; Born: UK; Ethnicity: White British).

“I feel like my soul is broken … Along with a traumatic birth all I wanted was a familiar hand to hold, a second pair of eyes and ears. I had never been so scared in my life, yet all it was, masked up strangers not wanting to come near you. It's a moment that will live with me for the rest of my life.” (Age: 24; Previous birth: No; Born: UK; Ethnicity: White British).

Mothers who went into labour spontaneously at home but planned to give birth in hospital were required to enter the hospital alone to be assessed, and those who arrived in advanced labour described the journey from hospital entrance to maternity unit as confused and frightening, particularly late at night where there was nobody to call on for assistance.

“When I got to hospital I was already in active labour, nearly fully dilated and I had to drag my bags on my own (partner wasn't allowed). It was quite late, the building looked empty and I felt that if I had started to deliver in elevator nobody would have noticed.” (Age: 38; Previous birth: Yes; Born: Poland; Ethnicity: Other White).

Women whose labour was started by induction generally faced much longer periods alone in hospital before they reached active labour and their birth partner was allowed in to support them. Being on their own, sometimes for several days of contractions, made this a more stressful and frightening start to their birth experience.

“I was in hospital alone for nearly 72 h being induced. The ward was a war zone, one lady gave birth in the corridor, screaming & crying … I was forgotten about. My experience is something I will seek help for when I fall pregnant again.” (Age: 29; Previous birth: No; Born: UK; Ethnicity: White British).

The distress was particularly intense where mothers had been disbelieved by health professionals as to the progress of their labour, leading to the unnecessary ongoing exclusion of their partners while they were in severe pain.

“I was preterm labour, scared + alone. Husband made it 6 min before 1st twin born. Unsupported—I have PTSD. Midwife wouldn't believe I was in labour as waters hadn't broken. Didn't think I was in enough pain. Wouldn't examine me after 3 cm despite requests—I was then fully dilated.” (Age: 34; Previous birth: No; Born: UK; Ethnicity: White British).

There were also some examples of women feeling well supported by professionals in the absence of their partners.

"Once I was in the hospital, surrounded by midwives and other women in my position (on our own until established labour), the care and attention from the midwives was second to none, they were fantastic, so caring, considerate and supportive.” (Age: 33; Previous birth: No; Born: UK; Ethnicity: White & Black Caribbean).

However, many mothers reported that midwives were too busy to give them adequate support during labour, as they were caring for many other mothers at the same time. Some mothers commented that even where professional support was available, this could not replace the emotional support of a chosen birth partner.

“I’m a 16 year old mum, I needed my mum there to get me through the needles / pain but was on my own until 5 cm dilated. I gave birth an hour later, I was having full blown contractions in a little room on my own. No midwifes there. It was awful.” (Age: 17; Previous birth: No; Born: UK; Ethnicity: White British).

“I was induced and the days leading up to the birth I was not allowed to have my birth partner with me which was devastating as I was alone at my most vunerable time. I needed someone to ressasure me, a familiar face. Even though the midwives were really nice I needed family with me.” (Age: 20; Previous birth: No; Born: UK; Ethnicity: Pakistani).

Although the survey did not ask questions about the impact on the mother’s partner, many mothers made use of the open text boxes to describe the emotional impact on their husbands, wives or partners of being denied the opportunity to be present during labour and sometimes during birth. This had caused them to feel anxious and helpless as they waited outside the hospital, sometimes for many hours and in cold temperatures, which had added to mothers’ own distress.

“Was unable to have my husband with me and I wasn’t informed this until I went into hospital. Really affected the both of us. I felt completely alone and he felt helpless.” (Age: 29; Previous birth: No; Born: UK; Ethnicity: White British).

#### Subtheme 3.3 Postnatal ward alone – coping with a newborn

When describing care after birth during a time when all visitors (including partners) were banned from postnatal wards, a small number of women said that they had received excellent care because they had felt practically and emotionally supported by staff.

“I was in pains every now and again with no family members support. It was quite a daunting experience. But with the supports from the midwives I was able to cope fairly with so much tears and bleeding in between.” (Age: 29; Previous birth: No; Born: Nigeria; Ethnicity: Black African).

Another positive reaction was that some liked being on the postnatal ward without being disturbed by other mothers’ visitors.

“I received great care in hospital and it was nice to have no partners walking about the maternity ward. It was good to wear knickers and no bra and not be embarrassed about males being present. The midwives focused just on us women and babies and no one else.” (Age: 29; Previous birth: No; Born: UK; Ethnicity: White British).

However, most mothers described the intense emotional strain of being separated from their partner or other companion shortly after birth (often within an hour). While accepting the principle that restricting visitors to postnatal wards was necessary for safety during the pandemic, they strongly believed that *‘birth partners aren’t visitors’*.

“Restrictions on birth partners post labour was wrong & deeply traumatic for every mother on postnatal ward. All you could hear was the sound of women crying.” (Age: 35; Previous birth: No; Born: United States; Ethnicity: White British).

“My husband was told to leave straight after the birth. The next time he saw me or his new baby was after 8 days. These were the hardest, most isolating 8 days of my life. It should not have been this way.” (Age: 36; Previous birth: No; Born: UK; Ethnicity: Pakistani).

As with other aspects of maternity care, many mothers described this enforced loss of emotional support as *‘frightening’*, particularly when their baby was unwell.

“I would like to mention how lonely giving birth in the pandemic is… Baby was born by elective section and then taken to NICU, and then dad had to go and I was on the ward on my own and my baby on a different ward which was terrifying.” (Age: 25; Previous birth: Yes; Born: UK; Ethnicity: White British).

Many women said they had found it hard to cope physically without the social support of their partner, mother and other visitors. They needed practical help with caring for their baby, and help with personal care such as showering or using the toilet; and they missed having someone else who could hold their baby while they looked after themselves. This was particularly difficult for mothers who were recovering from a difficult birth or a caesarean section, but also applied to mothers who were exhausted and in pain after a straightforward birth.

“I had to take care of a newborn baby for 2 days and a half basically on my own after a c-section. I couldn’t even take a shower because my baby wouldn’t settle at all, I had blood from the operation in my body and I felt alone, overwhelmed and desperate.” (Age: 36; Previous birth: No; Born: Spain; Ethnicity: Other White).

“Found ward after delivery v. difficult with no partner/help. Hadn't slept in 4 days, bleeding profusely, hadn't eaten in 30 h. Really struggled with baby on own without shower, toilet, food, water. Needed help!” (Age: 33; Previous birth: No; Born: UK; Ethnicity: White British).

Many mothers described the staff on postnatal wards as too busy to compensate for the absence of the practical and emotional support from partners or other family members. In particular, some highlighted feeling desperate because of extreme tiredness if their baby did not settle to sleep: they had to stay awake as was no one else to look after their baby.

“I stayed alone with the baby for 3 days after a caesarean. The nurses did not even for an hour take the baby away so I could sleep, thus I did not sleep for three days… very, very hard experience being all alone, no help, no support.” (Age: 37; Previous birth: No; Born: Israel; Ethnicity: Other White).

Mothers’ fears as they tried to stay awake were not hypothetical. There were some examples where mothers’ exhaustion and isolation, combined with a lack of support from staff, had created dangerous situations where mothers had been unable to keep their babies physically safe.

“My partner was only allowed to stay for 15 min after birth. Staff on ward said they would keep an eye on my baby + I, they didn't. I nodded off holding baby, my baby fell to the floor resulting in a fractured skull + bleed on the brain.” (Age: 37; Previous birth: Yes; Born: UK; Ethnicity: White British).

Some mothers said that their distress at being without any social support had been compounded by the unkind attitudes of staff on the postnatal ward who communicated indifference or hostility in response to mothers’ requests for help.

“My baby was born in midnight, but then we were sent to a dark room and not seen by anyone until 8 am … Mums opposite me were crying and asking for help as well, but no one is coming…My vagina was torn from giving birth, and I could barely walk. I asked for help to walk myself to the toilet but got rejected.” (Age: 29; Previous birth: No; Born: Hong Kong; Ethnicity: Chinese).

“The experience badly affected my mental status … Staff of postnatal department were very rude. They get so irritated if calling them by pressing bell … I wish I could have one family member beside me. Postnatal staff members could be a little kind to newborn and 1st time mum, a mum can have beautiful memories rather nightmare memories like me.” (Age: 32; Previous birth: No; Born: Bangladesh; Ethnicity: Bangladeshi).

Unsympathetic staff attitudes could also undermine the parenting confidence of mothers who were already conscious that they could not cope without support and felt that they were ‘failing’ on the very first days of their baby’s lives.

“Midwives seemed unaware of the full true impact of partners being missing from the ward. It made me feel so inadequate because I was expected to be coping as well on my own as I would have if my husband had been there… Even if the midwives are unable to do more, they could understand better and be less judgemental.” (Age: 38; Previous birth: No; Born: UK; Ethnicity: White British).

Some mothers focused on how enforced separation almost immediately after birth had harmed their ability to start life as a family, by undermining the processes of psychological adjustment and bonding for both parents.

“My partner was going to be told to leave the hospital until I pressed them to allow him an opportunity to meet and hold his newborn son. The midwife took the baby to his father who was told he had only 5 min to meet his child without me. After that he had to leave, giving us no chance to be a family, no skin-to-skin, and no goodbye after such a scary, painful event.” (Age: 27; Previous birth: No; Born: UK; Ethnicity: White British).

As well as delaying fathers and co-mothers from getting to know their new baby, it had left them worried about their partner, particularly after a difficult birth:

“When my husband was asked to leave the hospital in the corridor, he was leaving behind his 1 h old baby daughter and his wife who had just lost 1.5 l of blood and was still paralysed from the waist down. He found this really hard and sat in the hospital car park for a long time because he just couldn’t bring himself to drive away.” (Age: 38; Previous birth: Yes; Born: United States; Ethnicity: Other Mixed/Multiple).

Similarly to mothers describing the fear and pain of unsupported labour, some mothers used the strongest possible terms (*‘the worst night of my life’ ‘hell on earth’*) to describe their experiences of being deprived of social support immediately after birth and feeling trapped in a postnatal ward where they did not feel physically or psychologically safe.

“On the ward during lockdown was hell… No husband to help … I was starved, dehydrated, lonely, sleep deprived and it wasn’t my baby-related. It was like I was in isolation in prison. No music, no tv, no day light or fresh air. Just me and baby … No communication. No explanation. No daddy.” (Age: 37; Previous birth: Yes; Born: UK; Ethnicity: White British).

## Discussion

The qualitative findings from this large survey of new mothers in England have illustrated how the chaotic uncertainty, reduction of maternity and postnatal care, and restrictions on social support in hospitals and birth centres during the first wave of the COVID-19 pandemic all created additional stress for pregnant women and new mothers. At the same time, these changes undermined mothers’ access to the social support from their partner and family that might have enabled them to cope with this stress. Unpredictable, inconsistent and poorly communicated changes to the rules undermined mothers’ confidence in the maternity system, and its ability to provide safe care for themselves and their babies.

The findings of theme 1 (chaos) resonate with Mishel’s theory of uncertainty in illness [36], which has also been applied to pregnancy by Handley [37] and Hui Choi et al. [38]. Mishel highlighted how trust and confidence in the healthcare provider as a credible authority could reduce uncertainty and make it less likely that uncertainty would be appraised as threatening—this would be described as perception-focused coping in social support theory [11]. Mothers come into maternal care with a wide range of pre-existing stressors and psychological vulnerabilities [4]. In the context of the uncertainty and fear related to catching COVID-19 as well as the normal uncertainty of pregnancy, the unpredictable and inconsistent changes to maternity care had the potential to compound mothers’ anxieties by creating the impression that there was no longer a system in place that they could trust to keep them safe. This feeling of being forgotten by the system contrasts with the findings of a qualitative study of 23 mothers in London [39], in which mothers were reported to believe that pregnant women would be a priority group for health services during the pandemic who ‘were never going to be forgotten’.

Frontline healthcare workers were at increased risk of hospitalisation due to COVID-19 infection in the first wave of the pandemic [40], and a longitudinal survey found a high prevalence of severe stress, severe anxiety and probable post-traumatic stress disorder in their self-selecting sample (*n* = 7,840) of the nursing and midwifery workforce in the UK [41]. Many mothers in this study recognised that the maternity services were under severe pressure with staff shortages caused by illness or redeployment to critical nursing care, but there were profound differences in how they experienced the impact of this pressure. Where health professionals were not able to manage their own pandemic-related anxiety nor to communicate updates in a timely and accessible way, this caused worried mothers to lose trust in them as credible authorities. By contrast, some respondents had experienced health professionals making every effort to minimise disruption, communicate changes effectively and allay mothers’ fears. For these mothers, the health professionals’ actions and attitudes had shown that they could be trusted, and they felt secure *at a personal level* [42] despite the chaos *in the system*. Effective communication from health professionals is associated with reduced antenatal anxiety and increased self-care in normal times [43]; these findings emphasise how the way in which health professionals communicate with mothers during a crisis can profoundly affect the way that mothers experience crisis-related stress. This suggest that disaster-readiness for maternity services could include training that incorporates the good practice in calm and clear communication that some mothers described; creating advance guidance on the acceptable level of care for uninfected women during a pandemic [30]; and a plan to support the psychological wellbeing of health professionals so that they are in a position to generate security for others.

The findings in theme 2 (abandoned) illustrate the value placed by mothers on routine maternity and postnatal care as a source of reassurance, information and guidance. The cancellation or replacement of in-person appointments with remote consultations meant that mothers lost out on practical checks for themselves and their babies. In the antenatal period this had caused anxiety about the safe progress of their pregnancies, and postnatally it had caused additional stress about their own physical recovery and mental wellbeing as well as the health of their baby. This may have been reflected in the increased rates of self-identified anxiety reported in the National Maternity Survey 2020 compared with the same survey in 2018: 22% during pregnancy compared with 13% in 2018, and 39% postnatally compared with 29% in 2018 [24].

Drawing originally on her reflections on support needed in the transition to parenthood, Meleis conceptualised the role of the nurse as facilitating transitions, to support both health and a sense of wellbeing [44]. National guidance in England emphasises that the role of postnatal care is primarily about support for the transition to parenthood [45], and Walker et al. [46] identified the ability of mothers to connect with midwives as key to overcoming barriers to a successful transition. Particularly for first time mothers in this study, the cancellation of antenatal classes, combined with reduced postnatal midwifery care and the lack of access to health visitors’ clinics, created a perfect storm of feeling unprepared and overwhelmed by the responsibilities of early motherhood. Some felt that the briefer appointments and lack of face to face contact had undermined their ability to form trusting relationships with health professionals, and some were led to question whether postnatal care was really of any value if it had been felt appropriate to (apparently) withdraw it completely. Taken together, the findings in theme 2 indicate important learning for ‘normal’ times as well: maternity care is highly valued by mothers, but their trust is easily lost, and feelings of safety are undermined where health professionals focus on care as a technical service and do not pay attention to the meanings that mothers attach to these interactions. It also emphasises the importance mothers place on ‘expert’ assistance with the challenges of early motherhood, and is thus an argument that adequately-resourced preparation for parenthood and postnatal care may help to reduce the very high personal and social costs of poor perinatal mental health.

The findings in theme 3 (alone) demonstrate the devastating emotional impact on mothers of being separated from their closest social support during pregnancy, early labour and in the immediate postnatal period, and sometimes at birth as well. In contrast to the Canadian mothers surveyed by Groulx et al. [29], compulsory exclusion of partners from antenatal appointments could cause real distress, particularly where the mother felt vulnerable, or if it was a high risk pregnancy or ‘bad news’ was received at the appointment. The presence of one-to-one support from a chosen companion is of great importance to mothers’ experiences of birth [47] and in its absence, continuous support from a midwife or doula can improve physical outcomes [48]. There were positive examples of mothers in this study feeling well supported by health professionals during intrapartum and postnatal care in a hospital or birth centre, echoing the positive experiences reported in a qualitative study of 25 mothers in England and Scotland during the pandemic, in which mothers felt safe and supported in hospital and midwives actively cared for a baby after birth [49]. However, most of the responses were dominated by accounts of fear and distress as mothers laboured for long periods without a companion and with only intermittent support from overstretched midwives. There was further fear, distress, and feelings of failure and judgement when mothers then had to cope alone with the needs of a newborn baby while trying to manage self-care for their own post-birth bodies and the psychological adjustment to motherhood, in the unfamiliar environment of a postnatal ward, which some experienced as undermining and hostile. New mothers may be very sensitive to perceived as well as actual criticism [18], and the training of all staff who come into contact with mothers in the postnatal period should ensure that they have the understanding and skills to give care that is supportive and affirming, as well as safe and respectful [45].

Exclusion of partners and other family at this time both increased mothers’ stress because it undermined their ability to begin life as a family, and undermined their ability to cope with the stress of the immediate postnatal period by depriving them of the practical and emotional social support they longed for. Fathers commonly report feeling excluded or uncertain of their role in maternity care [50]. The findings of theme 3 underline the importance of seeing the mother and baby in their social context and providing care that is both mother-focused and family-centred [45]; in practical terms this includes recognising the mother’s partner or chosen companion as a vital resource for her social support throughout antenatal, intrapartum and postnatal care, whose presence and participation should be welcomed and encouraged at all times if the mother so chooses. This is essential to mothers’ wellbeing in ‘normal’ times, and should be seen as even more important during a crisis, not less.

The quantitative findings from the National Maternity Survey 2020 [24] indicated high rates of satisfaction with antenatal care (84%) and intrapartum care (85%), but much lower rates of satisfaction with postnatal care (53%). These qualitative findings suggest a more complex picture across the maternity period: mothers were keen to express gratitude for the work of NHS staff during the pandemic, and yet also experienced harm to their emotional wellbeing as a result of the systemic changes or the actions and attitudes of individual members of staff. Given the long-term cost of perinatal mental health difficulties in ‘normal’ times (estimated at £8.1 billion per annual cohort of births in the UK) [51], preparation for future crises should consider ways to protect the emotional wellbeing of mothers from additional stress.

### Strengths and limitations

This is the first qualitative analysis of maternity care experiences during COVID-19 using a survey based on random population-based sampling, building on earlier research in which open text responses to surveys with non-random samples (advertised on social media) were used to investigate experiences of maternity care, but not specifically emotional reactions, during the first wave of the pandemic in Canada, Republic of Ireland and USA [21, 29, 52]. LaDonna et al. [53] critiqued qualitative analysis of free-text responses in survey instruments on the grounds that these responses do not usually produce sufficiently rich data to yield rigorous qualitative results. On emotive topics, however, respondents may use open text comments to tell their story and communicate what they think is important [54]. In responding to the National Maternity Survey, many mothers described both their experiences and their emotional responses eloquently and/or at length. The challenges for this study came not from thinness of data but from its volume.

Women who responded to the National Maternity Survey were more likely to be older, born in the UK, and living in more advantaged areas of England, compared to those who did not respond [24]. However, the qualitative analysis of this large dataset prioritised responses from mothers who identified themselves as being from Black, Asian, and other minority ethnic backgrounds, and mothers who were younger, to foreground the experiences of women who might be more likely to experience disadvantage because of ethnicity, migration or age.

It was a limitation of this study that only one of the open text questions explicitly asked about impact, and it is possible that mothers who restricted themselves to factual descriptions of their experiences might have had fewer very positive or very negative reactions to their experiences. Qualitative analysis of open text is not a substitute for an in-depth interview where a participant’s account can be probed, but it can be a rich source of information where thousands of participants from diverse backgrounds describe emotionally charged experiences and many choose to describe their emotional reactions in detail.

## Conclusions

Mothers valued maternity care and some experienced additional stress from chaotic changes and reduction in care during the pandemic, as well as restrictions on essential social support during pregnancy, labour and birth. Planning for future crises should include considering how necessary adaptations to care can be implemented and communicated to minimise distress; ensuring that mothers are not deprived of social support at the time when they are at their most vulnerable; and supporting the psychological welfare of staff at a time of enormous pressure. There are also lessons for maternity care in ‘normal’ times: that care is highly valued, but trust is easily lost; that some mothers come into the maternity system with vulnerabilities that can be ameliorated or intensified by the attitudes of staff; that every effort should be made to welcome a mother’s partner or chosen companion into maternity and postnatal care, if she wishes; and that high quality postnatal care can make a real difference to the wellbeing of (particularly first time) mothers.

## Implications for pandemic- and broader disaster-readiness

- Create guidance now on the acceptable level of maternity care for uninfected mothers during a pandemic, and consistent protocols for care of infected mothers, with the goal of minimising distress as well as ensuring physical safety of staff and service users.
- Plan how to support the psychological wellbeing of maternity professionals so that they can generate psychological security for others.
- Train maternity professionals on calm and clear communication about necessary changes to care, to reduce maternal stress.
- Recognise social support as a key resource for protecting mothers’ emotional wellbeing, which may be even more important during a crisis.

## Data Availability

The datasets analysed during the current study are not publicly available due to the consent process but are available from the corresponding author on reasonable request.
